# A cumulative analysis of odontogenic cysts from major dental institutions of Bangalore city: A study of 252 cases

**DOI:** 10.4103/0973-029X.80030

**Published:** 2011

**Authors:** Prashanth Ramachandra, Prathima Maligi, HP Raghuveer

**Affiliations:** *Department of Oral Pathology, Dayananda Sagar College of Dental Sciences, Shavige Malleshwara Hills, Kumarswamy Layout, Bangalore, India*; 1 *Department of Oral and Maxillofacial Surgery, Dayananda Sagar College of Dental Sciences, Shavige Malleshwara Hills, Kumarswamy Layout, Bangalore, India*

**Keywords:** Dentigerous cyst, keratocyst, odontogenic cyst, radicular cyst

## Abstract

**Background:**

The objective of this study was to perform a cumulative analysis of odontogenic cysts obtained from the data of major dental institutions of Bangalore city, as well as to evaluate their distribution during a 5-year period and compare the results with other international studies.

**Materials and Methods:**

Data for the study were obtained from the reports of patients diagnosed with odontogenic cysts between 2005 and 2010 from different dental institutions of Bangalore. Case records of patients that fit the histological classification of the World Health Organization (WHO) (2005) were included in the study and the following variables were analyzed: age, gender, anatomic location, and histological type.

**Results:**

In a total of 252 cyst specimens diagnosed, 79.76% were odontogenic cysts and 20.24% were nonodontogenic cysts. Among the odontogenic cysts most frequent lesions were radicular cysts (50.25%), followed by keratocysts (27.36%) and dentigerous cysts (22.39%).

**Conclusions:**

Our study provides a cumulative data of odontogenic cysts in the population of Bangalore city. The results of our study showed a similar frequency of odontogenic cysts as compared to other populations of the world, with radicular cyst being identified as the most frequent odontogenic cyst. Keratocyst was the second most common cyst followed by dentigerous cyst.

## INTRODUCTION

A cyst is defined as “a pathological cavity having fluid, semi-fluid, or gaseous contents and which is not created by accumulation of pus”—Kramer 1974.[1] Most cysts in the jaw, with rare exceptions, are epithelial lined cysts and usually derived from odontogenic apparatus and remnants. These odontogenic cysts are encountered relatively commonly in dental practice.[2] According to the most recent WHO classification odontogenic cysts are classified into two main categories that reflect their pathogenesis. These are inflammatory cysts, such as radicular cysts, and developmental cysts, such as dentigerous and keratocysts.[3,4]

Commonly, odontogenic cysts exhibit slow growth and have a tendency towards expansion. Despite their benign biological behavior, these cysts can reach considerable size, if not diagnosed on time and treated appropriately.[5] The commonly encountered odontogenic cysts in all diagnostic oral pathology departments around the world include radicular cysts, dentigerous cysts, and odontogenic keratocysts.[4–6]

Radicular cysts are the most common cysts of the jaw, which have been classified as inflammatory cysts originating from epithelial cell rests of Malassez, secondary to pulpal necrosis. Dentigerous cysts enclose the crown of unerupted tooth and are attached to the neck of the tooth. The exact pathogenesis of dentigerous cyst remains unknown; however, it is believed to develop from a tooth follicle. Odontogenic keratocysts are clinically aggressive cystic lesions believed to arise from dental lamina or its remnants. The most characteristic clinical aspect of keratocyst is its high frequency of recurrence.[1]

Surprisingly, there are no statistical based studies of odontogenic cysts in South India, particularly from Bangalore city. The aim of this project was to carry out a clinico-pathological study of odontogenic cysts in Bangalore city and to analyze variables such as age, gender, site, and histological type, as well as to compare our findings with other studies from different geographical locations of the world.

## MATERIALS AND METHODS

A retrospective survey of odontogenic and nonodontogenic cysts was undertaken by the Department of Oral and Maxillofacial Pathology, Dayananda Sagar College of Dental Sciences, Bangalore. Data were cumulated from different leading dental teaching hospitals of Bangalore city. The records of 252 patients operated under general anesthesia or local anesthesia for cyst removal were included in the study. Data were retrieved from case notes and histopathology reports from March 2005 to March 2010. The data were analyzed for age, gender, histopathology (type of cyst), and anatomic location. Anatomic sites considered were maxilla and mandible, which were further divided into anterior, antero-posterior, and posterior regions. Categorization of all cysts was done as odontogenic cysts and others. Residual cysts were included along with radicular cysts. Eruption cysts were separated from dentigerous cysts. The *Microsoft Excel*^™^ software was used for data analysis and construction of graphs.

## RESULTS

During a 5-year period, a total of 252 cyst specimens were received from different leading dental teaching hospitals of Bangalore city. Table 1 shows the prevalence of odontogenic cysts and non-odontogenic cysts distributed by histological findings, gender, and age. Of these specimens, 201 cases (79.76%) were diagnosed as odontogenic cysts and 51 cases (20.24%) were diagnosed as nonodontogenic cysts. Overall odontogenic cysts were diagnosed more frequently in males 61.19%, with a male to female ratio of 1.58:1. The mean age for overall odontogenic cysts was 29.89 years. In relation to site, maxilla accounted for maximum number of cases (53.23%).

**Table 1:** Distribution of all cysts based on histopathology, gender and age of affected patients

Odontogenic cysts	Maxilla (n=107)			Mandible (n=94)		
	Anterior (%)	Anteroposterior (%)	Posterior (%)	Anterior (%)	Anteroposterior (%)	Posterior (%)
Radicular	55 (83.33)	7 (10.60)	4 (6.60)	11 (31.42)	7 (20.0)	17 (48.57)
Keratocysts	15 (71.42)	1 (4.76)	5 (23.80)	13 (38.23)	1 (2.94)	20 (58.82)
Dentigerous	13 (65.0)	3 (15.0)	4 (20.0)	7 (28.0)	0 (0.0)	18 (72.0)
Total	83 (77.57)	11 (10.28)	13 (12.15)	31 (32.98)	8 (8.51)	55 (58.45)

**Figure 1:**
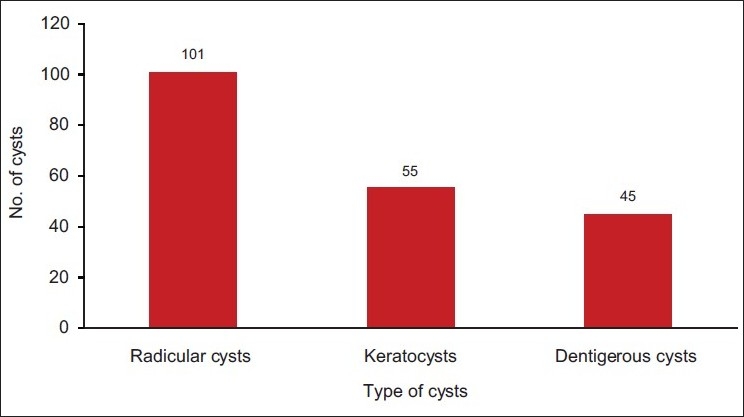
Distribution of odontogenic cysts according to number

Among 201 odontogenic cysts the largest diagnostic group was the radicular cyst, which accounted for 101 cases (50.25%), with a male to female ratio of 1.81:1 and mean age of 30.56 years at the time of diagnosis [Figure 1]. In the second largest group accounting for 55 odontogenic keratocysts (27.36%), 34 were diagnosed in males and 21 in females, with a male to female ratio of 1.62:1 and a mean age of 29.36 years. The dentigerous cyst represented the next set of lesions, accounting for 45 cases (22.39%) with a male to female ratio of 1.14:1 and a mean age of 29.77 years. Among nonodontogenic cysts, epidermoid and dermoid cysts accounted for maximum number of cases.

**Figure 2:**
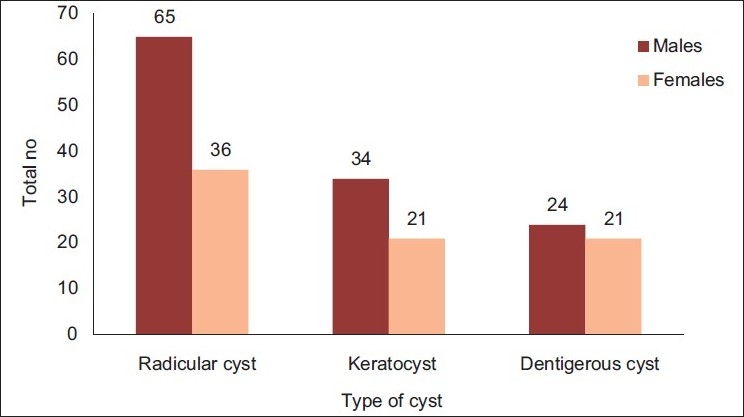
Distribution of odontogenic cysts between both sexes

Figure 2 depicts the distribution by age (decades) of the three major groups of odontogenic cysts observed in our study. Although radicular cysts, odontogenic keratocysts, and dentigerous cysts were observed in the first decade of life, but the peak was seen in the third decade for all cysts with progressive decline.

**Figure 3:**
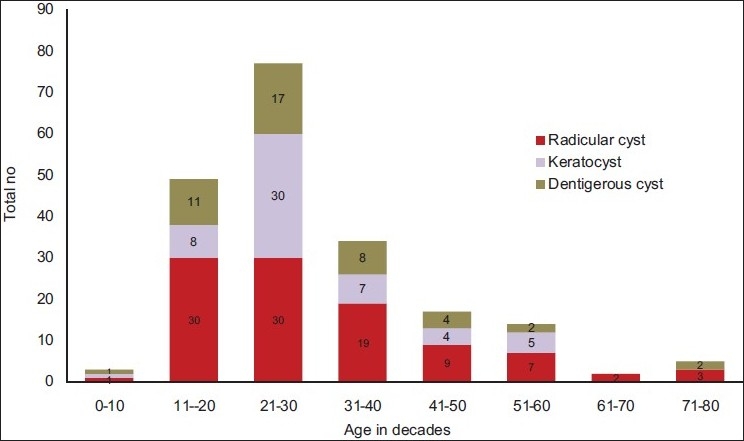
Distribution of odontogenic cysts in different decades of age

**Figure 4:**
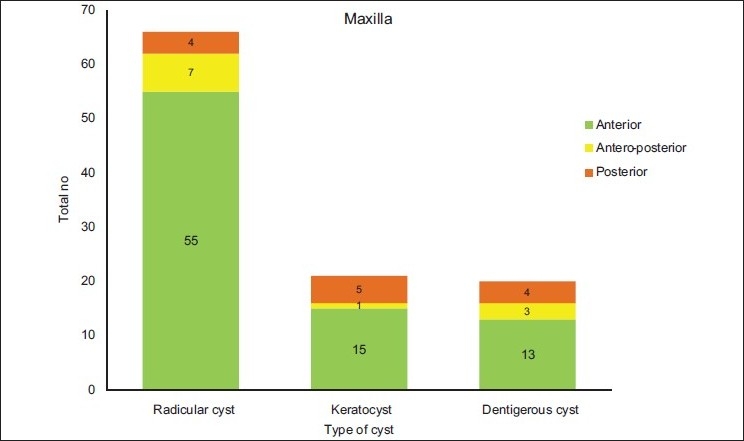
Distribution of odontogenic cysts according to anatomic site in the maxilla

**Figure 5:**
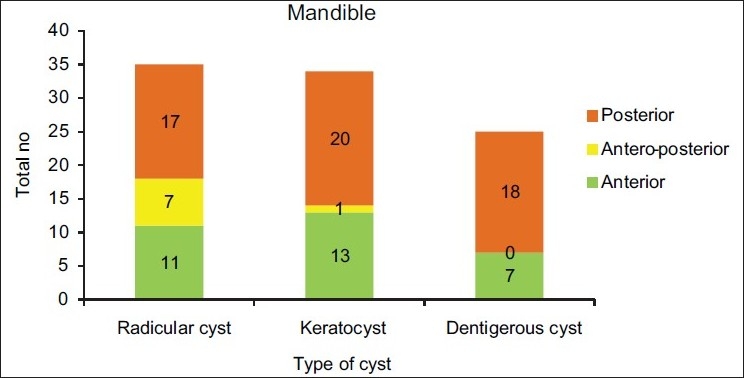
Distribution of odontogenic cysts according to anatomic site in the mandible

Table 2 and Figures 3–5 show the anatomical location of the three common odontogenic cysts in the maxilla or mandible. Radicular cysts were prevalent in the anterior part of maxilla with 83.33% cases. In the mandible, posterior region showed maximum number of cases (48.57%). Odontogenic keratocysts were more common in the posterior mandible region (58.82%) and in the maxilla; anterior maxilla was the common site (71.42%). Dentigerous cysts were slightly more prevalent in the mandible compared to maxilla, with posterior mandible accounting for maximum number of cases (72.0%).

**Table 2:** Distribution of odontogenic cysts according to anatomic site

S. No.	Type of cyst	Total (%)	Male (%)	Female (%)	Sex ratio (M:F)	Mean age (±SD)

	Odontogenic cysts	201 (79.76)	123 (61.19)	78 (38.81)	1.58	30.29 (±14.74)
1.	Radicular	101 (50.25)	65 (64.36)	36 (35.64)	1.81	30.92 (±15.91)
2.	Keratocyst	55 (27.36)	34 (61.82)	21 (38.18)	1.62	29.55 (±11.86)
3.	Dentigerous	45 (22.39)	24 (53.33)	21 (46.67)	1.14	29.78 (±15.33)

	**Non-Odontogenic cysts**	**51 (20.24)**	**36 (70.59)**	**15 (29.41)**	**2.4**	**28.81 (±13.02)**

1.	Epidermoid cyst	22 (43.13)	17 (77.27)	5 (22.72)	3.4	34.55 (±12.59)
2.	Dermoid cyst	13 (25.49)	9 (69.23)	4 (30.77)	2.25	25.00 (±7.35)
3.	Mucocele	11 (21.57)	7 (63.64)	4 (36.36)	1.75	20.91 (±10.09)
4.	Eruption cyst	3 (5.88)	2 (66.67)	1 (33.33)	2	26.67 (±4.16)
5.	Nasolabial cyst	2 (3.92)	1 (50.00)	1 (50.00)	1	52.50 (±7.78)
	Total	252 (100)	159 (63.09)	93 (36.90)	1.70	30.29 (±14.71)

## DISCUSSION

Odontogenic cysts constitute frequent benign lesions of jaw bones, due to the ubiquitous presence of epithelial rests after odontogenesis. Most of these cysts share similar clinical and radiographic features; therefore, their diagnosis should be based on the careful examination of clinical, radiographic, and histopathological features.[6] The most reliable sources for obtaining information relative to the frequency, clinical–radiographical, and histological characteristics of these lesions are from the department of oral pathology, where the biopsies are processed.

This study examined the distribution and correlation of common odontogenic cysts from leading dental teaching hospitals of Bangalore city during the 5-year period (March 2005 – March 2010). In general, the prevalence and distribution of odontogenic cysts in Bangalore showed some similarity to that reported in various other studies. Odontogenic cysts were diagnosed in 7-12% of all oral and maxillofacial biopsies, which is in agreement with the findings of our study (9.80%).[7,8] In the present study, odontogenic cysts occurred in adult men more frequently than in women, and these findings are in accordance with those of other studies.[5–7,9–12] Maxilla was the most commonly affected site, which is in agreement with other similar studies by Ochsenius **et al**.,[5] Procktaa **et al**.,[8] Bataineh *et al*.,[11] and Varinauskas *et al*.,[13] But this differs from the findings of Meninguad *et al*.,[10] Koseoglu *et al*.,[12] and Avelar *et al*.,[14] where the mandible was the most common site.

Radicular cysts are lesions produced as the consequences of pulpal necrosis and are therefore considered to be inflammatory in nature. In the present study, radicular cyst accounted for 50.25% of all odontogenic cysts and was the most frequent of all odontogenic cysts. This finding is similar to that described by Tay[15] who reported a frequency of 50.7% and the same as that described by Ochsenius *et al*.,[5] 50.7%, Mosqueda *et al*.,[7] 52% and Shear[16] 52.3%. The prevalence among the male gender was slightly higher (64.36%; M:F = 1.81) than the female gender in the present study, which is in agreement with the findings from various other studies[6,10,12] and in disagreement with some studies.[5,9]

The greater prevalence among the male gender in some studies may be explained by the fact that men usually have poor oral hygiene and are more susceptible to trauma than woman. According to the literature, most frequently affected site is the anterior maxilla,[5,6,9,11,12] which is in agreement with our findings but also differs from those reported by Meningaud *et al*.[10] and Avelar *et al*.,[14] They have reported that mandible was the most commonly affected site. The fact that anterior region of the maxilla is the most frequently affected site may be explained by esthetic factors, as the patients may wish to preserve their anterior teeth without adequate restorative endodontic treatment. Peak incidence of radicular cysts was seen in the second and third decades of life with a gradual decline from fourth decade onwards in our study, and this finding is in agreement with other studies.[6,12]

With respect to dentigerous cyst this lesion occupied the third place with the relative frequency of 22.39%, which was in agreement with Tay’s study where dentigerous cyst occupied the third place with a frequency of 15.2%.[15] But in most other studies, dentigerous cysts occupied the second place.[7,16] Males showed a slightly greater predilection compared to females (53.33%; M:F = 1.14), a finding that was in accordance with other studies.[5,14] Dentigerous cysts occurred over a wide age range with a peak incidence in the second and third decades of life, which corroborates the findings of the previous studies.[8,9,14] Pertaining to site predilection, the mandibular third molar region was the most common site of presentation accounting for 72% of all known sites of occurrence. The next most frequently affected site was the anterior maxillary canine region. This finding was similar to the those of various other studies,[5,6,11,12] but differed from Waldron, who found that the upper third molar to be the most prevalent site.[17] This is perhaps not a surprising finding, given the fact that lower third molar and upper canines are the teeth most commonly affected by impaction.

According to the new World Health Organization (WHO) classification, odontogenic keratocysts have now been reclassified as keratocystic odontogenic tumors.[18] For comparison, we have maintained the previous classification system.[3] With the inclusion of keratocyst, it occupied the second position in our study with a frequency of 27.36% similar to that of Koseoglu *et al*.[12] (27%) and in contrast to other studies such as Daley *et al*.[19] (4.88%), Nakamura *et al*.[20] (7.7%), and Shear[16] (11.2%). In relation to gender, we observed a moderate predominance of males (61.82%; M:F = 1.62), which other authors have also referred to a similar findings.[5,7] Regarding age distribution, highest incidence was seen in the third decade; these findings coincide with those of Ochsenius *et al*.[5] and Ahlfors *et al*.[21] These are most often seen in the mandible, with a strong predilection for the molar ramus region, and our study also showed similar results.[18,20,22–25]

## CONCLUSIONS

As this is a retrospective study, it has all drawbacks intrinsic to this type of studies, for example, it is not always possible to find the necessary data in the case history, renew the lost information; besides, the interpretation of data may be improper. We compared the results of our study with various other populations around the world and found that inflammatory cysts are identified as the most frequently occurring odontogenic cyst. Further studies should be performed in different regions of Indian sub-continent and world populations in order to determine the global epidemiological profile of these lesions.
